# Reliable Reference Genes for Normalization of Gene Expression in Cucumber Grown under Different Nitrogen Nutrition

**DOI:** 10.1371/journal.pone.0072887

**Published:** 2013-09-13

**Authors:** Anna Warzybok, Magdalena Migocka

**Affiliations:** Wrocław University, Institute of Experimental Biology, Department of Plant Molecular Physiology, Wroclaw, Poland; University of Pittsburgh, United States of America

## Abstract

In plants, nitrogen is the most important nutritional factor limiting the yield of cultivated crops. Since nitrogen is essential for synthesis of nucleotides, amino acids and proteins, studies on gene expression in plants cultivated under different nitrogen availability require particularly careful selection of suitable reference genes which are not affected by nitrogen limitation. Therefore, the objective of this study was to select the most reliable reference genes for qPCR analysis of target cucumber genes under varying nitrogen source and availability. Among twelve candidate cucumber genes used in this study, five are highly homologous to the commonly used internal controls, whereas seven novel candidates were previously identified through the query of the cucumber genome. The expression of putative reference genes and the target *CsNRT1.1* gene was analyzed in roots, stems and leaves of cucumbers grown under nitrogen deprivation, varying nitrate availability or different sources of nitrogen (glutamate, glutamine or NH_3_). The stability of candidate genes expression significantly varied depending on the tissue type and nitrogen supply. However, in most of the outputs genes encoding CACS, TIP41, F-box protein and EFα proved to be the most suitable for normalization of *CsNRT1.1* expression. In addition, our results suggest the inclusion of 3 or 4 references to obtain highly reliable results of target genes expression in all cucumber organs under nitrogen-related stress.

## Introduction

Real-time quantitative reverse transcription polymerase chain reaction (RT-qPCR) is currently the method of choice for mRNA transcription studies, since it provides outputs with high sensitivity, specificity and capacity [1], [2]. However, for accurate gene expression quantification, it is essential to normalize real-time PCR data to a fixed reference. Reference genes are commonly referred to as genes of highly reliable expression, which is not affected by various experimental settings and is stable in different types of tissues and organs used in the assay [3]. The most widely used internal controls include the genes encoding: actin and tubulin (alpha/beta), cytoskeletal proteins; glyceraldehyde 3-phosphate dehydrogenase (GAPDH), involved in glycolysis; ubiquitins (UBQs), involved in the degradation of cellular proteins; 18S RNA, a part of the ribosomal functional core; RNA polymerase II (RPII or POLR2A), catalyzing the synthesis of the precursors of mRNA, most snRNA and microRNA; elongation factor 1-alpha (EF1α), which facilitates translational elongation; tyrosine-3 monooxygenase/tryptophan-5 monooxygenase activation protein; zeta polypeptide (*YWHAZ*), and hypoxanthine phosphoribosyl transferase 1 (HPRT1), which has a central role in the generation of purine nucleotides [4]–[13]. Nevertheless, growing evidence clearly shows that the expression of genes commonly used as reliable internal controls is often significantly affected during different experimental conditions [14]. Consequently, the systematic evaluation of common and novel reference genes derived from genome-wide analyses is becoming an essential component of real-time reverse transcription–PCR analysis so as to improve the reliability of published results [3], [15], [16]. Recently, a number of statistical algorithms have been developed to evaluate the expression stabilities of candidate reference genes in order to select the most reliable reference for a particular experimental assay. GeNorm [14], NormFinder [17] and BestKeeper [18] are free VBA (Visual Basic Applets) applets for Microsoft Excel that have been commonly used to determine the expression stability of candidate reference genes in plants such as potato [19]; grapevine [20]; rice [21], [22]; tomato [23], [24]; soybean [25]; citrus [26]–[28]; coffee [29]; brachiaria grass [30]; peach [31]; cotton [32]; eucalyptus [33]; chicory [34]; cucumber [35], [36]; petunia [37]; banana and plantains [38]; and peanut [39]. As a result, some suitable references were selected for studying gene expression analyses in a variety of differentially developed plant tissues and organs or in plants grown under biotic and abiotic stresses. However, there is currently little knowledge about the expression stability of commonly used reference genes or novel references under varying nitrate availability or different sources of nitrogen in the environment. Nitrogen is an important constituent of the majority of essential structural, genetic and metabolic compounds in plant cells [40], [41]. It is a structural component of all amino acids, the building elements of structural and enzyme proteins, and chlorophyll, the pigment essential for photosynthesis. In addition, nitrogen builds the energy-transfer compounds, such as ATP, electron-transfer molecules, such as NAD(P) or FAD, and the genetic material essential for growth and reproduction (nucleic acids DNA and RNA). Hence lack or deficiency of the available nitrogen in soil solution results in severe disturbances in the synthesis and action of key cellular biomolecules, causing a detrimental effect on plant growth and development [40]–[42]. In soil solution, the majority of plant-available nitrogen exists in the inorganic NH_4_^+^ and NO_3_^−^ forms, but most crop plants require mainly nitrate in large quantities to sustain high yields [43], [44]. In contrast to ammonium, the level of nitrate in soils is highly variable, since NO_3_^−^ ions do not bind to soil complexes and thus leach easily when excess water percolates through the soil [45]. Numerous studies have been performed to reveal mechanisms that underlie the plant response to nitrogen or nitrate deprivation or varying availability. The estimation of patterns of expressed genes may provide insight into complex regulatory networks and help to identify genes involved in the signaling and metabolic pathways underlying developmental and cellular processes in plants grown under different nitrogen nutrition.

Since the accurate quantification of the expression of genes involved in nitrogen transport or metabolism requires reliable internal controls with highly stable expression independent of nitrogen or nitrate supply and concentration, the selection of suitable candidate genes is highly desired. We have recently identified seven novel candidates for reference genes in cucumber encoding :CACS (clathrin adaptor complex subunit), PDF2 (protein phosphatase 2), TIP41 (PPA2 activator), GW881873 (expressed protein), F-box, HEL (helicase), YSL8 (mitosis protein) and analyzed the expression of novel genes as well as the five commonly used references: *ACT (actin)*, *TUA (tubulin)*, *CYP (cyclophilin)*, *UBI-1* (*ubiquitin*), *EFα (elongation factor α*) under abiotic stress and plant growth regulators [36]. In this work, we present for the first time the genomic organization of all 12 analyzed reference genes in cucumber whole genome contigs and validate their expression stability under varying nitrate supply and various nitrogen sources. Based on the obtained results, we propose reliable internal controls for studies of the expression of cucumber genes involved in nitrogen transport and metabolism.

## Materials and Methods

### Plant material

Cucumber plants were grown essentially as described earlier [46], with slight modification of nitrogen source and availability in nutrition media. The roots, stems or leaves were collected from 2-week-old plants grown under different source of nitrogen and from 4-week-old plants grown upon different nitrate provision. Two-week-old plants were grown for the first week in media containing 5 mM KNO_3_, and for the second week they were transferred to the fresh N-free nutrient solution containing K_2_CO_3_ instead of KNO_3_. Following nitrogen starvation, plants were transferred to the fresh N-free medium or to the media enriched in 5 mM KNO_3_, 5 mM NH_4_Cl, 5 mM glutamine (Gln) or 5 mM glutamic acid (Glu) for 6 or 12 hours. Part of the 4-week-old plants were grown on N-free medium or media containing 0.5 mM or 10 mM KNO_3_ for 4 weeks. Some of the plants were transferred for one week into 0.5 mM KNO_3_ following 3 weeks of growth in N-free medium (temporary nitrate provision). Other plants were grown for the first 2 weeks in N-free medium, then for one week in 0.5 mM KNO_3_-containing medium and for the last week again in N-free medium (temporary nitrate starvation). Last part of the seedlings was grown as follows: plants grown for the first week in N-free medium and for the second week in 0,5 mM nitrate were again put into N-free medium for the third week and transferred into fresh medium containing 0,5 mM nitrate for the last (fourth) week (temporary nitrate re-supply). All nutrient solutions were aerated and changed three times a week. For each treatment four samples (50 mg) of each tissue from four different plants were collected and stored at −80°C until use.

### Total RNA isolation and cDNA synthesis

Total RNA was extracted using the TRI-Reagent (Sigma) according to the protocol provided by manufacturer. RNA quantity and quality was assessed spectrophotometrically (Nanodrop) and the samples showing A260/A280 ratio of 1.8–2.0 and an A260/A230 ratio of 2.0–2.2 were used for subsequent analysis. RNA integrity was assessed on an Agilent 2100 Bioanalyzer using the RNA 6000 pico labchip Kit (Agilent Technologies). All samples were further treated with RNase-free DNase (Fermentas), according to the manufacturer's instructions. First strand cDNA was synthesized by reverse transcribing 2 µg of total RNA with high-capacity cDNA Reverse Transcription kit (Applied Biosystems, USA) in a 20 µl reaction using random primers and MultiScribe™ Reverse Transcriptase (50 U) according to manufacturer's instructions. Reverse transcription was performed at 37°C for 1 hour followed by 85°C for 5 min. cDNA was diluted 8 times for the use of real-time qRT-PCR reaction. All cDNA were stored at −20°C until use.

### The organization and validation of reference genes

We have previously selected 12 putative reference genes and validated their expression stability in cucumber roots or roots, stems and leaves under abiotic stress (heavy metals, salt, osmotic and oxidative stress) and different plant growth regulators [36]. They included five references commonly used in the studies on plant genes expression (*actin, tubulin, cyclophilin, ubiquitin* or *elongation factor*) as well as seven putative candidates homologous to novel reference genes selected in *A. thaliana* [3]: *clathrin adaptor complex subunit CACS (At5g46630*), *expressed protein At33380*, *TIP41(At54g34270), helicase (At1g58050)*, *phoshpolipase 2 PDF2 (Atg13320)*, *mitosis protein YSL8 (At5g08290)* and *F-box (At5g15710)*. The full sequences of all cucumber genes were identified in the whole-cucumber genome shotgun reads using the queries of *A. thaliana* cDNAs, Blastn [47] and FGENESH or FGENESH+ [48] softwares. The genomic organization and putative function of all selected candidate genes are presented in table 1. The gene encoding cucumber nitrate transporter NRT1.1 was used as the target for the normalization of expression data. Primer pairs on the selected reference and target gene sequences (Table S1) were designed using the Lightcycler Probe Design software (Roche), with the conditions of 154–290 base pairs (bp) as the PCR amplicon length and 60°C as the optimal Tm (melting temperature).

**Table 1:** Description of cucumber candidate reference genes based on the comparison with their Arabidopsis orthologs.

Gene symbol and lenght	NCBI contig accession no	Gene position within contig	NCBI EST accession no	Arabidopsis ortholog locus	Arabidopsis ortholog description	Function
ACT 1134 bp	ACHR01010658	7140–8587	AB010922	At5g09810	Actin 7	Structural constituent of cytoskeleton, protein binding
TUA 1467 bp	ACHR01012753	92843–95078	AJ715498	At4g14960	Tubulin alpha-6	Structural constituent of cytoskeleton, protein folding
EFα 1344 bp	ACHR01002194	9646–11514	EF446145	At5g60390	Elongation factor 1-alpha	Translational elongation
CYP 519 bp	ACHR01007623	9364–9882	AY942800	At2g16600	Peptidyl-prolyl cis-trans isomerase CYP19-1	Protein folding, signal transduction
CACS 1278 bp	ACHR01010524	38791– 44675	GW881874	At5g46630	AP-2 complex subunit mu-1 (Clathrin adaptor complexes medium subunit family protein)	Intracellular protein transport, vesicle-mediated transport
HEL 1014 bp	ACHR01008789	1228–5426	GW881869	At1g58050	RNA helicase family protein	Helicase activity
TIP41 873 bp	ACHR01001003	28762–31836	GW881871	At4g34270	TIP41-like family protein	PP2A phosphatase activator
UBI-1 306 bp	ACHR01007578	26294–27611	AF104391	At5g57860	Ubiquitin family protein	Protein binding, protein modification
F-box 1378 bp	ACHR01005017	38102–39873	GW881870	At5g15710	F-box/kelch-repeat protein	Unknown
YSL8 429 bp	ACHR01006572	23112–24359	GW881872	At5g08290	Yellow-Leaf-Specific gene 8, YLS8	Vesicle-mediated transport, mitosis, vacuole and Golgi organization
GW881873 984 bp	ACHR01016153	59983–63343	GW881873	At4g33380	Expressed protein	Unknown
PDF2 2103 bp	ACHR01000299	57230–62778	GW881868	At1g13320	Protein Phosphatase 2A Subunit A3	N-terminal protein myristoylation, regulation of phosphorylation

Seven of the twelve candidate cucumber reference genes (*CACS*, *TIP41*, *PDF2*, *GW881873*, *YSL8*, *HEL*) have been recently retrieved from the whole cucumber genome sequence [36] using the novel reference genes identified in *Arabidopsis* as the query sequences [3]. The commonly used remaining five genes (*ACT*, *TUA*, *UBI-1*, *EFα*, *CYP*) were previously available in the Genbank database as partial cDNAs. The full cDNAs and exon/intron organization of all 12 candidate genes were established using BlastN, and FGENESH or FGENESH+.

### Amplification of gene transcripts

The expression study was performed using a 96 well plate on an Lightcycler 480 (Roche) with 2× SYBR Green Mix B (A&A Biotechnology). The reactions were performed according to the manufacturer's instructions: the PCR program was initiated at 95°C for 10 min to activate *Taq* DNA polymerase, followed by 45 thermal cycles of 10 seconds at 94°C, 10 seconds at 60°C and 15 seconds at 72°C. Melting curve analysis was performed immediately after the real-time PCR. The temperature range used for the melting curve generation was from 65°C to 95°C. All assays were performed using three technical and biological replicates, a non-template control and a non-RT control. The standard curves were generated by amplifying at least seven dilution series of cDNA (Table S1). The correlation coefficient (R^2^) and PCR efficiency were calculated using the slopes of the standard curves (Figure S2). The linear R^2^ for all the primers ranged between 0.978–0.999, whereas PCR efficiencies of primers ranged from 95%–105% (Figure S2, Table S1). To confirm the PCR products size, the reactions were subjected to electrophoresis on 2.0% agarose gels stained with ethidium bromide following Real-time PCR assay. The determination of the crossing amplification point (Cp) as well as the relative quantification analysis (ΔΔCT-method) were performed using the Lightcycler 480 software 1.5. The amplification of non-template controls generated Cp values above 45 or was not detectable. The non-normalized expression data were analyzed by geNorm v3.5 and NormFinder version 2 whereas the raw Cp values were imported into BestKeeper version 1.

### The evaluation of reference gene expression stability

Considering the heterogeneity of treatments, the biological samples from 2-week-old plants and 4-week-old plants were analyzed separately. For each analysis of stability of gene expression, four subsets were established based on the organ used, including roots, stems, leaves and all organs of cucumber plants. At first, the reliability of all twelve cucumber candidate genes was evaluated using two different statistical algorithms, geNorm [14] and NormFinder [17]. Based on the their outputs, the two worst references were removed and the expression stability of the remaining ten genes was further validated using BestKeeper [18]. All three Visual Basic applets for Microsoft Excel base on different principles. The geNorm calculates an internal control gene-stability measure *M* as the average pairwise variation of each gene with other candidate genes and select two ideal references through the sequential exclusion of genes with the lowest stability of expression [14]. The lower the *M* value, the higher stability of the expression of particular gene. In addition, geNorm calculates the optimal number of genes required for normalization of target gene expression based on the pairwise variation (V_n/n+1_) between normalization factors established for defined number of reference genes. The inclusion of additional reference is not required if the V value is below the 0,15 [14]. Contrary to geNorm, the NormFinder calculates and ranks the stability value for each gene investigated based on the comparison between the intra-group and inter-group variations of candidate genes expression [17]. The lowest stability value correspond to the highest expression stability, but a minimum of 3 genes and a minimum of 2 samples per group are required for the analysis. Similar to geNorm and Normfinder, the Bestkeeper estimates the most appropriate reference by using the geometric mean of the expression of the candidate cDNA, however, it takes into account the raw data instead of the data converted in relative quantity. In addition, a maximum 10 candidate genes may be analyzed using this applet. BestKeeper calculates a pairwise correlation coefficient between each gene and the BestKeeper index (BI) and a standard deviation (SD) of the Cp-values between the whole data set. The gene with the most stable expression should have the highest coefficient of correlation with the BI indicates [18].

## Results

### The genomic organization and expression of cucumber reference genes

12 candidate reference genes were identified from two sources: traditional housekeeping genes *actin, ubiqutin, cyclophilin, tubulin, elongation factor α*, frequently used for transcript normalization in cucumber were found in the GenBank database whereas cucumber homologues to the superior reference genes selected from *Arabidopsis* transcriptome microarray data [3]: *clathrin adaptor complex subunit CACS, expressed protein GW881873*, *TIP41, helicase*, *phoshpolipase 2 PDF2*, *mitosis protein YSL8* and *F-box*, were found within the cucumber whole-genome contigs available in GenBank under the accession number ACHR0100000. The accession numbers of genes and contigs, gene names and length as well as the functions of putative proteins according to The Arabidopsis Initiative Resource (TAIR) are listed in table 1. Real-time PCR analysis of the transcript abundance revealed that the particular candidate reference genes displayed different expression ranges across the full set of cucumber samples assayed (Figure S1). While the avarage C_p_ values varied from 17 with a SD±1.5 for *EFα* gene to 26 with SD±2.0 for *PDF2*, most of the genes analyzed have shown an expression rate between 19 and 24. The genes encoding CYP, YSL8 and TUA showed the most variations in expression between all samples assayed, whereas the Cp values of *CACS*, *EFα* and *F-box* genes were more uniformly expressed. Taken together, all twelve genes displayed a relatively wide range of expression levels in cucumber roots, stems and leaves.

### Expression stability analysis

**Figure 1:**
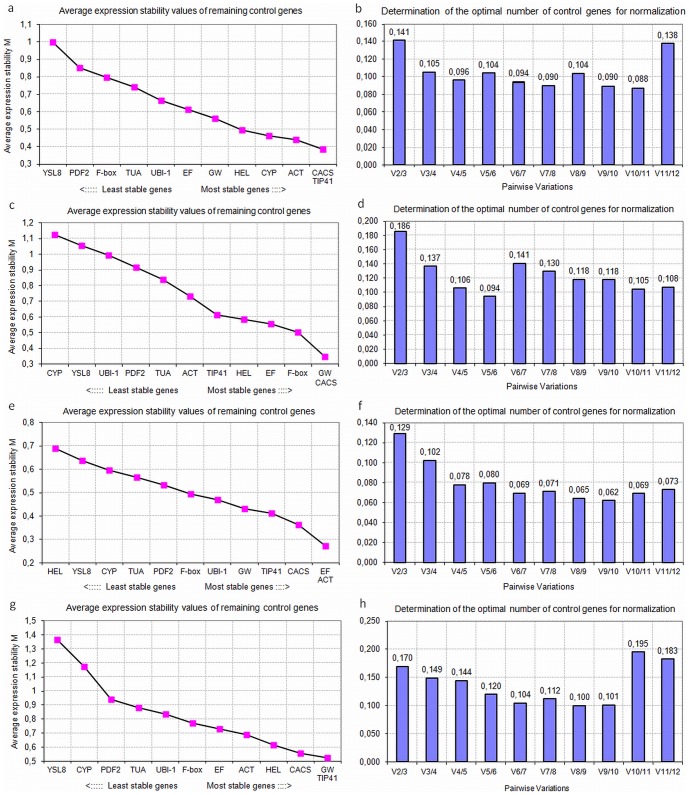
GeNorm based evaluation of candidate gene expression in samples from plants grown in different nitrogen compounds. Average expression stability values (*M*) of the remaining candidate cucumber reference genes during stepwise exclusion of the least stable reference gene in roots (a), stems (c), leaves (e) and all cucumbers organs taken together (g). The lowest the M values indicate the most stable expression of candidate cucumber genes. Determination of optimal number of reference genes based on pairwise variation (V) analysis of normalization factors of the candidate reference genes in roots (b), stems (d), leaves (f) and all cucumber organs taken together (h). The V_n/n+1_ value was calculated for every comparison between two of the twelve consecutive candidate reference genes. According to [14], additional (n+1)^th^ reference gene should be included into analysis whenever the V_n/n+1_ value drops below the 0.15 threshold.

**Figure 2:**
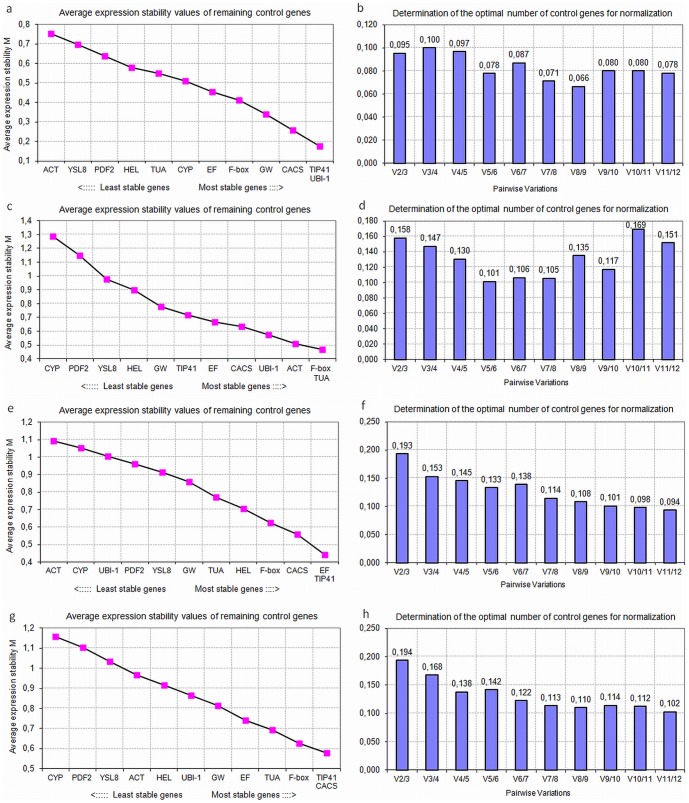
GeNorm based evaluation of candidate gene expression in samples from plants grown in different nitrate supply. Average expression stability values (*M*) of the remaining candidate cucumber reference genes during stepwise exclusion of the least stable reference gene in roots (a), stems (c), leaves (e) and all cucumbers organs taken together (g). The lowest the M values indicate the most stable expression of candidate cucumber genes. Determination of optimal number of reference genes based on pairwise variation (V) analysis of normalization factors of the candidate reference genes in roots (b), stems (d), leaves (f) and all cucumber organs taken together (h). The V_n/n+1_ value was calculated for every comparison between two of the twelve consecutive candidate reference genes. According to [14], additional (n+1)^th^ reference gene should be included into analysis whenever the V_n/n+1_ value drops below the 0.15 threshold.

In order to find the most suitable internal control for cucumber RT-qPCR normalization, we assessed the stability of expression of 12 candidate genes using the pairwise variation in expression stability implemented in geNorm v3.5 as well as the NormFinder, which estimates the stability of gene expression based on the comparison between inter- and intra-group variability. The samples from 2-week-old plants and 4-week-old plants were analyzed separately with regard to the different developmental stage and differential treatment. Figures 1 and 2 show the *M* values of reference genes examined by geNorm when the samples were considered separately as roots, stems and leaves or together, as all organs. The outputs revealed some significant differences between particular candidate genes expression stability in individual cucumber organs and under different nitrogen nutrition. In roots, *CACS*, *TIP41*, *ACT* and *CYP* showed the most stable expression upon different nitrogen sources, whereas *YSL8* and *PDF2* ranked at the worst positions (Figure 1a and 2a). In contrast, *ACT* was the least stable gene in roots grown under varying nitrate, where in turn, beside *CACS* and *TIP41*, the expression of *UBI-1* gene was very constant (Figure 2a). More significant variations were observed in stems, were *CACS, GW881873* and *F-box* or *ACT, F-box* and *TUA* were ranked at top positions in samples from plants grown under different nitrogen source or varying nitrate availability, respectively (Figure 1c and 2c). On the contrary, *CYP, YSL8* and *PDF2* were ranked poorly in stem tissues regardless the nutritional treatment, whereas *UBI-1* showed an uniform expression under differential nitrogen treatment (M<0.6) but was less stable under varying NO_3_^−^ supply (M∼1) (Figure 1c and 2c). The differential treatment of plants also affected the ranking of gene expression in leaves, where *EFα, TIP41* and *CACS* were the most reliable genes but either *HEL, YSL8* and *CYP* or *ACT, CYP* and *UBI-1* showed the least stable expression under varying nitrogen source or nitrate supply, respectively (Figure 1e and 2e). Like in roots, in leaves *ACT* was ranked as the best gene upon differential nitrogen nutrition and as the worst gene under varying nitrate supply (Figure 1e and 2e). Despite some apparent differences in all rankings generated by geNorm, the overall analysis of candidate genes expression in all samples from roots, stems and leaves confirmed that *TIP41* and *CACS* ranked at top positions whereas *YSL8, PDF2* and *CYP* ranked as the most variable genes regardless the heterogeneity of plant treatment (Figure 1g and 2g). Though all of the 12 genes showed acceptable expression stabilities (*M*≤1.5), according to Vandesompele et. al. [14], the *M* values were general higher when samples from all organs were analyzed together (Figure 1 and 2). In addition, geNorm also calculated the optimal number of reference genes required for a more reliable normalization (V_n/n+1_). Taking into account the entire dataset from 2-week-old plants and considering a cut-off (V_n/n+1_≤0.15), the pairwise value for two genes (V_2/3_) was 0.17, while for three genes (V_3/4_) was 0.149 (Fig. 1h). Therefore, the third reference gene should be included for normalization to improve further gene expression evaluations in cucumbers grown under different nitrogen compounds. The overall analysis of gene expression in 4-week-old plants revealed the V_2/3_ and V_3/4_ values of 0.194 and 0.168, respectively, whereas the pairwise value for four genes (V_4/5_) was 0.138 (Figure 2h). Based on the analysis, a minimum of four references would be necessary for accurate analysis of target genes expression in plants grown under varying nitrate supply. Similarly to geNorm, NormFinder determined *TIP41*, *CACS*, *EFα*, or *F-box* as the most stable reference genes in the two entire datasets, whereas *CYP*, *PDF2* or *YSL8* were usually ranked as the most variable (Table 2). Commonly used *actin* was again ranked in the last positions when samples from roots or all organs from plants grown on varying nitrate were considered, however, it displayed relatively stable expression in roots and leaves of plants grown in different nitrogen compounds (Table 2). The highest variability in candidate gene expression reflected by the highest stability values calculated by NormFinder was observed when all organs from plants grown in different nitrogen source were analyzed together (Table 2). Further evaluation of the ten most stable reference genes in BestKeeper confirmed that *CACS*, *TIP41*, and *F-box* were ranked in the highest positions in samples from 2-week-old plants (Table 3). *CACS* was also the most reliable gene in stems, leaves and all organs taken together in plants grown on varying nitrate, whereas *EFα* was better in roots (Table 4). Although the results obtained by the three algorithms seem to be a bit divergent in the rankings of candidate genes, they show that at least four sufficiently reliable reference genes (*CACS*, *TIP41*, *F-box*, *EFα*) could be suitable for normalizing all cucumber sample sets. Moreover, the geNorm-based overall analysis of the expression of twelve candidate genes in all samples collected from 2-week-old and 4-week-old cucumbers grown under different nitrogen source and varying nitrate availability revealed, that *CACS*, *TIP41*, *F-box* and *EFα* show the highest expression stability not only in conditions of various nitrogen nutrition but also at different developmental stages of cucumber plants (Figure S3).

**Table 2:** Candidate cucumber genes ranked according to their expression stability as determined by NormFinder.

Ranking order	Plants grown under different source of nitrogen*				Plants grown under varying availability of nitrate**			
	Roots	Stems	Leaves	All organs	Roots	Stems	Leaves	All organs
**1**	***TIP41***	***TIP41***	***EF***	***CACS***	***EF***	***CACS***	***CACS***	***CACS***
	0.016	0.114	0.147	0.248	0.160	0.268	0.074	0.180
**2**	***CACS***	***F-box***	***CACS***	***TIP41***	***CACS***	***Tubulin***	***EF***	***EF***
	0.086	0.144	0.169	0.253	0.171	0.326	0.121	0.273
**3**	***Actin***	***CACS***	***Actin***	***F-box***	***F-box***	***UBQ***	***TIP41***	***TIP41***
	0.094	0.156	0.192	0.281	0.197	0.346	0.122	0.289
**4**	***F-box***	***GW881873***	***UBQ***	***EF***	***TIP41***	***TIP41***	***Tubulin***	***GW881873***
	0.102	0.166	0.193	0.331	0.198	0.359	0.225	0.311
**5**	***EF***	***EF***	***GW881873***	***GW881873***	***UBQ***	***GW881873***	***F-box***	***F-box***
	0.130	.182	0.210	0.367	0.209	0.362	0.243	0.341
**6**	***GW881873***	***Helicase***	***TIP41***	***Actin***	***GW881873***	***EF***	***Actin***	***UBQ***
	0.169	0.193	0.241	0.385	0.235	0.366	0.323	0.342
**7**	***Cyclophilin***	***TUA***	***PDF2***	***UBQ***	***Cyclophilin***	***F-box***	***Helicase***	***Tubulin***
	0.186	0.228	0.249	0.433	0.247	0.376	0.324	0.378
**8**	***UBQ***	***Actin***	***F-box***	***PDF2***	***Tubulin***	***Helicase***	***YSL8***	***Helicase***
	0.208	0.257	0.266	0.455	0.295	0.378	0.331	0.391
**9**	***Tubulin***	***Cyclophilin***	***Cyclophilin***	***Helicase***	***Helicase***	***YSL8***	***UBQ***	***PDF2***
	0.264	0.315	0.351	0.459	0.298	0.383	0.341	0.416
**10**	***Helicase***	***PDF2***	***Tubulin***	***Tubulin***	***YSL8***	***Actin***	***PDF2***	***YSL8***
	0.295	0.334	0.380	0.615	0.334	0.430	0.361	0.451
**11**	***PDF2***	***YSL8***	***Helicase***	***YSL8***	***PDF2***	***Cyclophilin***	***GW881873***	***Actin***
	0.326	0.335	0.412	1.158	0.380	0.655	0.384	0.452
**12**	***YSL8***	***UBQ***	***YSL8***	***Cyclophilin***	***Actin***	***PDF2***	***Cyclophilin***	***Cyclophilin***
	0.521	0.351	0.444	1.269	0.426	0.741	0.479	0.479

Stability values are listed from the most stable to the least stable gene.

* Samples from two-week-old plants grown under nitrate, ammonia, glutamine or glutamate for 4 or 12 hours.

** Samples from 4-week-old plants grown under nitrogen deficiency, 0.5 mM nitrate, 10 mM nitrate, temporary nitrate provision, temporary nitrate starvation or temporary nitrate re-supply.

**Table 3:** BestKeeper based evaluation of reference genes stability in cucumber plants grown in different nitrogen compounds.

Gene	UBQ	EF	ACT	GW881873	F-BOX	CYP	TIP41	CACS	HEL	TUA	PDF2	YSL8
**Roots**	0,819	0,890	0,894	0,837	0,929	0,934	**0,981**	0,951	0,834	0,820	-	-
**Stems**	-	0,980	0,964	0,965	**0,989**	0,614	0,979	0,972	0,972	0,861	0,931	-
**Leaves**	0,969	0,983	0,974	0,976	0,950	0,939	0,966	**0,985**	**-**	0,873	0,981	-
**All organs**	0,855	0,969	0,930	0,954	0,904	-	**0,981**	0,973	0,916	0,817	0,776	-

The stability values were calculated based on the pairwise correlation between genes and BI (BestKeeper Index). The highest Person coefficient values representing the most stable genes are marked in bold. Genes ranked at the lowest positions by geNorm and NormFinder for each set of analyzed samples were not included (-) in BestKeeper evaluation.

**Table 4:** BestKeeper based evaluation of reference genes stability in cucumber plants grown in varying NO_3_^−^ supply.

Gene	UBQ	EF	ACT	GW881873	F-BOX	CYP	TIP41	CACS	HEL	TUA	PDF2	YSL8
**Roots**	0,706	**0,989**	-	0,908	0,940	0,919	0,893	0,986	0,919	0,924	-	0,835
**Stems**	0,810	0,949	0,760	0,963	0,986	-	0,983	**0,992**	0,941	0,989	-	0,982
**Leaves**	0,889	0,994	-	0,942	0,986	-	0,990	**0,996**	0,970	0,952	0,931	0,923
**All organs**	0,701	0,779	-	0,886	0,790	-	0,956	**0,988**	0,824	0,740	0,963	0,950

The stability values were calculated based on the pairwise correlation between genes and BI (BestKeeper Index). The highest Person coefficient values representing the most stable genes are marked in bold. Genes ranked at the lowest positions by geNorm and NormFinder for each set of analyzed samples were not included (-) in BestKeeper evaluation.

## Discussion

Investigation of the expression level of genes encoding enzymes and proteins involved in nitrogen transport and metabolism is the crucial step in understanding the mechanisms underlying plant response to nitrogen supply, which could help breeders to improve crop fertilization and production. The availability of cucumber genomic resources [49], [50] allows for identification of the whole families of genes involved in nitrogen metabolism and nitrogen compounds' uptake, transport and assimilation in this plant. To date, only two studies relying on RT-qPCR analysis in cucumber have validated candidate reference genes for transcript normalization. These studies included a few test conditions such as abiotic stresses (salinity, drought, osmotic and oxidative stress, heat, cold) and plant growth regulators [35], [36]. Still, the evaluation of reference genes expression in cucumber grown under different nutritional conditions is lacking.

Here, we evaluated the stability of expression of seven novel and five traditional reference genes in roots, stems and leaves of cucumbers grown under different nitrogen regimes. Initial analysis in geNorm and NormFinder showed some differences in the particular rankings of candidate genes; however, both applets consistently selected similar genes as showing the most stable or unstable expression patterns. The slight divergences probably result from different basic assumptions of the geNorm and NormFinder models. The NormFinder estimates both the intra- and inter-group variation to calculate a stability value for each candidate gene. The lower the inter- and intra-group variations, the higher the position of the gene in the ranking. In contrast, geNorm selects the best two internal control genes taking into account only similar intergroup variation, which may be problematic in the case of co-regulated genes [51]. Differences between NormFinder and geNorm outputs were also demonstrated by other studies [28], [29], [35], [36], [52], [53]. The best 10 reference genes were further analyzed by BestKeeper, which calculated the coefficient of variance of each putative reference gene as a percentage of the average Cp level. Based on the three different outputs, all algorithms seem to be relevant to elect internal controls for each experimental set. In all cases, the best reference genes recommended by one program were also highly ranked by the two others. Our results demonstrated that *CACS*, *TIP41*, *EFα*, and *F-box* were the most stably expressed reference genes in most samples and subsets studied. Nevertheless, the best combination of genes varied significantly depending on experimental condition and organ assayed. This observation confirms that the validation of the stability of candidate genes expression is a prerequisite for reliable normalization in specific biological samples and assays. Among top ranked genes, *TIP41* and *EFα* were identified as the most stable genes in roots, whereas *EFα* and *CACS* were uniformly expressed in leaves of cucumber plants. Beside *F-box*, *CACS* and *TIP41* were also highly ranked in stems. Our results support the previous study on cucumber candidate reference genes, demonstrating that *CACS*, *TIP41*, *F-box* and *EFα* showed the most stable transcript accumulation under heavy metal, salt, osmotic or oxidative stress and upon application of growth regulators [36]. Wan et.al. [35] also demonstrated that *EFα* expression in cucumber was highly stable in different tissues and under abiotic and biotic stress. In addition, Czechowski et.al. [3] also observed that genes homologous to the cucumber top references *EFα*, *CACS* and *F-box* were stably expressed in *Arabidopsis* roots under abiotic stress. Moreover, *CACS*, *TIP41*, *F-box* and *EFα* were among the most reliable candidate references in samples from different developmental stages, organs, tissues and genotypes of *Arabidopsis* plants (M≤0.5), as calculated by geNorm software [3]. Interestingly, *EFα* expression was significantly affected by S, P or sugar starvation in *Arabidopsis* [3] whereas its cucumber homolog was stably expressed upon nitrogen starvation. The gene encoding elongation factor was also top ranked during nitrogen starvation in tomato [24]. It may be cautiously concluded that the expression of genes encoding elongation factors is not significantly altered upon nitrogen deficiency, so they could serve as suitable internal controls in conditions of nitrogen-related stress. In the case of *F-box*, beside *Arabidopsis* [3], [54] and cucumber [36], this gene was also considered a good candidate gene for normalizing a wide range of tissue from soybean and citrus or floral organs in cotton [25], [28], [32]. To date, F-box proteins have been shown to participate in ubiquitination of the proteins targeted for degradation, signal transduction and cell cycle regulation [55]. Such basic physiological functions are usually maintained by constitutively expressed housekeeping genes. Although the F-box protein identified in cucumber has not been functionally characterized yet, it seems to be a constitutively expressed gene in the whole plant regardless of nitrogen availability and source, and thus could be recommended as a reliable reference for studying target genes expression under nitrogen-related stress. Similarly to *F-box*, *YSL8* was also ranked among the top reference genes in various differentially developed organs of *Arabidopsis* and in roots and leaves of thale cress treated with elevated Cu and Cd [3], [54]. In contrast, *YSL8* was ranked among the least stably expressed candidate genes under different nitrogen nutrition (Figure 1 and 2, Table 2). Similarly, the gene was usually ranked in a lower position in roots or in roots, stems and leaves of cucumbers grown under abiotic stress and phytohormones [36]. Hence, we may conclude that *YSL8* may not be a suitable reference gene in studies of cucumber gene expression. Similarly to *YSL8*, *Clathrin adaptor complex subunit* is also involved in intracellular and vesicle-mediated transport; however, *CACS* was ranked in the top positions in all analyses in our study, regardless of cucumber organs identity or treatment. Clathrin adaptor proteins link clathrin to their receptors in vesicles, forming a coat, which is important for cargo selection and direction of the vesicle transport [56]. Endocytosis and exocytosis of vesicles are performed by cells to take up nutrients, to import signaling receptors or to mediate export of toxic compounds [57]. Perhaps the expression of *CACS* remains constitutive regardless of nutritional condition because the protein encoded by this gene participates in such basic, intracellular transport processes. The other novel cucumber candidate reference was *PDF2*, which was uniformly expressed in the *Arabidopsis* organs at various developmental stages [3]. However, in the previous studies on cucumber, *PDF2* displayed intermediate or low stability values in plants grown with application of phytohormones and abiotic stress [36]. In the current analyses *PDF2* along with *YSL8* was considered the least stable candidate gene under different nitrogen nutrition. *PDF2* is one of three genes encoding the 65 kDa regulatory subunit of protein phosphatase 2A (PP2A), which plays crucial roles in the regulation of growth and development [58]. Altered PP2A activity in plants was associated with disturbances in hormone homeostasis and signaling, defense responses, cell division, morphogenesis, and reproduction [59]. Since the growth and development of plant is significantly affected by nitrogen availability, such a regulatory protein may not be a suitable reference for normalization of organ samples from plants grown under a varying nitrogen source or supply. The last candidate reference among novel cucumber genes was *HEL*, which was found to be very stable in a series of developmental samples and different organs in *Arabidopsis* [3]. In cucumber, *HEL* was ranked in intermediate positions when samples from plants grown under abiotic stress, phytohormones [36] or nitrogen-related stress (Figure 1 and 2, Table 2) were analyzed. Therefore, a better reference could be suggested for studies of target genes expression in cucumber.

Genes commonly referred to as housekeeping genes, such as *tubulins*, *actins*, *cyclophilins* or *ubiquitins*, have often been used as internal controls in target genes expression studies. Validation of expression stability of these genes in plants has brought contradictory results. Czechowski et. al. [3] found *ACT2* to be the least stably expressed gene in *Arabidopsis* among the 27 samples from different stages, organs and conditions. *ACT* was also considered unreliable during flax development [53] and citrus grown in drought stress [28]. In cucumber, the expression of *ACT7* homolog was found to be significantly affected during abiotic stress and phytohormone treatment [36]. Though the gene was ranked generally in lower positions during nitrogen-related stress, it was found among the most reliable genes in roots or leaves of plants grown in different nitrogen sources (Figure 1, Table 2). *Actins* were also considered highly stable genes in cucumber grown in cold or heat (*ACT3*, *ACT2*, *ACT1*), drought or salt (*ACT2*, *ACT3*), hormones (*ACT2*) or when the samples from different tissues or treatments were taken together (*ACT*, *ACT3*) [35].

Similarly to *ACT*, *TUB* was also ranked in the last position for different genotypes of citrus analyzed in various experimental conditions [27], [28] and during flax development [53]. In cucumber, *TUA* was considered an inadequate reference gene under heavy metals, oxidative, salt and osmotic stress [36] as well as under high or low temperature [35]. However, the expression of the gene was highly stable when different cucumber tissues or samples from plants treated only with three different hormones – ABA (abscisic acid), SA (salicylic acid) and MeJA (methyl jasmonic acid) – were analyzed [35]. In our study, *TUA* was ranked mostly in an intermediate position in all subsets analyzed, except for the stems from plants grown in varying nitrate supply, where it displayed significantly higher expression stability. Given the observations, both *actins* and *tubulins* can be considered adequate internal controls for expression studies on target genes in cucumber in particular conditions.

Similarly to *YSL8* and *PDF2*, *CYP* was ranked among the least stable candidate genes in cucumber grown under different nitrogen nutrition. It was also considered an unreliable internal control in cucumbers grown under abiotic stress and phytohormones [35], [36]. The expression of *CYP* also significantly varied in different tissues of peach and maize, in potato grown in abiotic and biotic stress, in grapevine during berry development and during wheat endosperm development [19], [20], [31], [60]. Although this gene was stably expressed in wheat flag leaves sampled in organic and conventional fields [61], it may not be a reliable internal control for expression analyses in cucumber plants.

The last common reference gene evaluated in cucumber was *UBI-1*, encoding the putative ubiquitin peptide which marks proteins to ensure their proper localization or degradation, as reviewed by [62]. Though generally ranked in intermediate positions, *UBI-1* demonstrated highly stable expression in roots and stems of plants grown under varying nitrate supply. *UBI-1* was also previously ranked among the top 5 candidate reference genes in cucumbers grown under heavy metals, oxidative, salt and osmotic stress [36] and subject to cold, heat or phytohormones [35]. Nevertheless, *UBI-1* expression appears to be less stable when compared to other candidates for reference genes.

In summary, despite slight differences found in different subsets, we concluded that at least four genes – *CACS*, *TIP41*, *EFα* and *F-box* – appear to be good reference genes for normalizing a wide range of organ samples of cucumber in different experimental conditions, even though the molecular and biological function of three of the putative proteins encoded by these genes (CACS, TIP41 and F-box) remains unclear. Moreover, our study suggests that more than two reliable internal controls should be used to normalize target genes expression in all cucumber organs under different nitrogen nutrition. In contrast, two reference genes were suitable for analysis of samples taken from plants grown under abiotic stress and phytohormones [35], [36]. Commonly used reference genes like *actins*, *tubulins* or *cyclophilins* and *ubiquitins* should be carefully evaluated for each experimental condition tested, since their expression may be significantly affected depending on organ identity or experimental assay. This work constitutes the first systematic study in cucumber to validate optimal reference genes for RT-qPCR normalization with consideration of different organs, various nitrogen source and varying NO_3_^−^ availability.

## Supporting Information

Figure S1 The average expression levels with SD of candidate reference genes in roots, stems and leaves of cucumber plants grown under different nitrogen nutrition.
(DOC)

Figure S2 **The parameters of real-time PCR amplification of candidate reference genes.**
(DOC)

Figure S3 GeNorm based evaluation of candidate gene expression in samples from plants grown in different nitrogen compounds or under varying nitrate availability. A. Average expression stability values (*M*) of the remaining candidate cucumber reference genes during stepwise exclusion of the least stable reference gene all cucumbers organs. The lowest the M values indicate the most stable expression of candidate cucumber genes. B. Determination of optimal number of reference genes based on pairwise variation (V) analysis of normalization factors of the candidate reference genes all cucumber organs. The V_n/n+1_ value was calculated for every comparison between two of the twelve consecutive candidate reference genes. According to [14], additional (n+1)^th^ reference gene should be included into analysis whenever the V_n/n+1_ value drops below the 0.15 threshold.
(DOC)

Table S1 **Primer sequences used to quantify the expression of the selected traditional and novel cucumber reference genes by real-time PCR.**
(DOC)

## References

[1] Bustin SA, Benes V, Nolan T, Pfaffl MW (2005) Quantitative real-time RT-PCR – a perspective. J Mol Endocrinol 34: 597–601. 15956331 10.1677/jme.1.01755

[2] Bustin SA, Benes V, Garson JA, Hellemans J, Huggett J, et al (2009) The MIQE guidelines: minimum information for publication of quantitative real-time PCR experiments. Clin Chem 55: 611–622. 19246619 10.1373/clinchem.2008.112797

[3] Czechowski T, Stitt M, Altmann T, Udvardi MK, Scheible WR (2005) Genome-wide identification and testing of superior reference genes for transcript normalization in Arabidopsis. Plant Physiol 139: 5–17. 16166256 10.1104/pp.105.063743 PMC1203353

[4] Thellin O, Zorzi W, Lakaye B, De Borman B, Coumans B, et al (1999) Housekeeping genes as internal standards: use and limits. J Biotechnol 75: 291–295. 10617337 10.1016/s0168-1656(99)00163-7

[5] Catalan V, Gomez-Ambrosi J, Rotellar F, Silva C, Rodriguez A, et al (2007) Validation of endogenous control genes in human adipose tissue: relevance to obesity and obesity-associated type 2 diabetes mellitus. Horm Metab Res 39: 495–500. 17611901 10.1055/s-2007-982502

[6] de Jonge HJ, Fehrmann RS, de Bont ES, Hofstra RM, Gerbens F, et al (2007) Evidence based selection of housekeeping genes. PLoS One 2: e898. 17878933 10.1371/journal.pone.0000898 PMC1976390

[7] Maroufi A, Bockstaele EV, Loose MD (2010) Validation of reference genes for gene expression analysis in chicory (Cichorium intybus) using quantitative real-time PCR. BMC Mol Biol 11: 15–27. 20156357 10.1186/1471-2199-11-15 PMC2830926

[8] Mehta R, Birerdinc A, Hossain N, Afendy A, Chandhoke V, et al (2010) Validation of endogenous reference genes for qRT-PCR analysis of human visceral adipose samples. BMC Mol Biol 11: 39. 20492695 10.1186/1471-2199-11-39 PMC2886049

[9] Tea M, Michael MZ, Brereton HM, Williams KA (2013) Stability of small non-coding RNA reference gene expression in the rat retina during exposure to cyclic hyperoxia. Mol Vis 19: 501–508. 23441123 PMC3580969

[10] Hu R, Fan C, Li H, Zhang Q, Fu YF (2009) Evaluation of putative reference genes for gene expression normalization in soybean by quantitative real-time RT-PCR. BMC Mol Biol 10: 93. 19785741 10.1186/1471-2199-10-93 PMC2761916

[11] Garg R, Sahoo A, Tyagi AK, Jain M (2010) Validation of internal control genes for quantitative gene expression studies in chickpea (Cicer arietinum L.). Biochem Biophys Res Commun 2: 283–288. 10.1016/j.bbrc.2010.04.079 20399753

[12] Uddin MJ, Cinar MU, Tesfaye D, Looft C, Tholen E, et al (2011) Age-related changes in relative expression stability of commonly used housekeeping genes in selected porcine tissues. BMC Research Notes 4: 441–454. 22023805 10.1186/1756-0500-4-441 PMC3219825

[13] Gu YR, Li MZ, Zhang K, Chen L, Jiang AA, et al (2011) Evaluation of endogenous control genes for gene expression studies across multiple tissues and in the specific sets of fat- and muscle-type samples of the pig. J Anim Breed Genet 128: 319–325. 21749479 10.1111/j.1439-0388.2011.00920.x

[14] Vandesompele J, De Preter K, Pattyn F, Poppe B, Van Roy N, et al (2002) Accurate normalization of real-time quantitative RT-PCR data by geometric averaging of multiple internal control genes. Genome Biol 3: RESEARCH0034. 12184808 10.1186/gb-2002-3-7-research0034 PMC126239

[15] Faccioli P, Ciceri GP, Provero P, Stanca AM, Morcia C, et al (2007) A combined strategy of “in silico” transcriptome analysis and web search engine optimization allows an agile identification of reference genes suitable for normalization in gene expression studies. Plant Mol Biol 63: 679–688. 17143578 10.1007/s11103-006-9116-9

[16] Paolacci AR, Tanzarella OA, Porceddu E, Ciaffi M (2009) Identification and validation of reference genes for quantitative RT-PCR normalization in wheat. BMC Mol Biol 10: 11. 19232096 10.1186/1471-2199-10-11 PMC2667184

[17] Andersen CL, Jensen JL, Orntoft TF (2004) Normalization of real-time quantitative reverse transcription-PCR data: a model-based variance estimation approach to identify genes suited for normalization, applied to bladder and colon cancer data sets. Cancer Res 64: 5245–5250. 15289330 10.1158/0008-5472.CAN-04-0496

[18] Pfaffl MW, Tichopad A, Prgomet C, Neuvians TP (2004) Determination of stable housekeeping genes, differentially regulated target genes and sample integrity: BestKeeper–Excel-based tool using pair-wise correlations. Biotechnol Lett 26: 509–515. 15127793 10.1023/b:bile.0000019559.84305.47

[19] Nicot N, Hausman JF, Hoffmann L, Evers D (2005) Housekeeping gene selection for real-time RT-PCR normalization in potato during biotic and abiotic stress. J Exp Bot 56: 2907–2914. 16188960 10.1093/jxb/eri285

[20] Reid KE, Olsson N, Schlosser J, Peng F, Lund ST (2006) An optimized grapevine RNA isolation procedure and statistical determination of reference genes for real-time RT-PCR during berry development. BMC Plant Biol 6: 27. 17105665 10.1186/1471-2229-6-27 PMC1654153

[21] Jain M, Nijhawan A, Tyagi AK, Khurana JP (2006) Validation of housekeeping genes as internal control for studying gene expression in rice by quantitative real-time PCR. Biochem Biophys Res Commun 345: 646–651. 16690022 10.1016/j.bbrc.2006.04.140

[22] Li QF, Sun SSM, Yuan DY, Yu HX, Gu M, et al (2010) Validation of candidate reference genes for the accurate normalization of real-time quantitative RT-PCR data in rice during seed development. Plant Mol Biol Rep 28: 49–57.

[23] Exposito-Rodriguez M, Borges AA, Borges-Perez A, Perez JA (2008) Selection of internal control genes for quantitative real-time RT-PCR studies during tomato development process. BMC Plant Biol 8: 131. 19102748 10.1186/1471-2229-8-131 PMC2629474

[24] Lovdal T, Lillo C (2009) Reference gene selection for quantitative real-time PCR normalization in tomato subjected to nitrogen, cold, and light stress. Anal Biochem 387: 238–242. 19454243 10.1016/j.ab.2009.01.024

[25] Libault M, Thibivilliers S, Bilgin DD, Radwan O, Benitez M (2008) Identification of four soybean reference genes for gene expression normalization. Plant Genome 1: 44–54.

[26] Liu Q, Zhu A, Chai L, Zhou W, Yu K, et al (2009) Transcriptome analysis of a spontaneous mutant in sweet orange [Citrus sinensis (L.) Osbeck] during fruit development. J Exp Bot 60: 801–813. 19218315 10.1093/jxb/ern329 PMC2652045

[27] Carvalho K, de Campos MK, Pereira LF, Vieira LG (2010) Reference gene selection for real-time quantitative polymerase chain reaction normalization in “Swingle” citrumelo under drought stress. Anal Biochem 402: 197–199. 20363209 10.1016/j.ab.2010.03.038

[28] Mafra V, Kubo KS, Alves-Ferreira M, Ribeiro-Alves M, Stuart RM, et al (2012) Reference genes for accurate transcript normalization in citrus genotypes under different experimental conditions. PLoS One 7: e31263. 22347455 10.1371/journal.pone.0031263 PMC3276578

[29] Cruz F, Kalaoun S, Nobile P, Colombo C, Almeida J, et al (2009) Evaluation of coffee reference genes for relative expression studies by quantitative real-time RT-PCR. Mol Breeding 23: 607–616.

[30] Silveira ED, Alves-Ferreira M, Guimaraes LA, da Silva FR, Carneiro VT (2009) Selection of reference genes for quantitative real-time PCR expression studies in the apomictic and sexual grass Brachiaria brizantha. BMC Plant Biol 9: 84. 19573233 10.1186/1471-2229-9-84 PMC2717968

[31] Tong Z, Gao Z, Wang F, Zhou J, Zhang Z (2009) Selection of reliable reference genes for gene expression studies in peach using real-time PCR. BMC Mol Biol 10: 71–84. 19619301 10.1186/1471-2199-10-71 PMC3224724

[32] Artico S, Nardeli SM, Brilhante O, Grossi-de-Sa MF, Alves-Ferreira M (2010) Identification and evaluation of new reference genes in Gossypium hirsutum for accurate normalization of real-time quantitative RT-PCR data. BMC Plant Biol 10: 49. 20302670 10.1186/1471-2229-10-49 PMC2923523

[33] Boava LP, Laia ML, Jacob TR, Dabbas KM, Goncalves JF, et al (2010) Selection of endogenous genes for gene expression studies in Eucalyptus under biotic (Puccinia psidii) and abiotic (acibenzolar-S-methyl) stresses using RT-qPCR. BMC Res Notes 3: 43. 20181283 10.1186/1756-0500-3-43 PMC2854107

[34] Maroufi A, Van Bockstaele E, De Loose M (2010) Validation of reference genes for gene expression analysis in chicory (Cichorium intybus) using quantitative real-time PCR. BMC Mol Biol 11: 15. 20156357 10.1186/1471-2199-11-15 PMC2830926

[35] Wan H, Zhao Z, Qian C, Sui Y, Malik AA, et al (2010) Selection of appropriate reference genes for gene expression studies by quantitative real-time polymerase chain reaction in cucumber. Anal Biochem 399: 257–261. 20005862 10.1016/j.ab.2009.12.008

[36] Migocka M, Papierniak A (2011) Identification of suitable reference genes for studying gene expression in cucumber plants subjected to abiotic stress and growth regulators. Mol Breeding 28: 343–357.

[37] Mallona I, Lischewski S, Weiss J, Hause B, Egea-Cortines M (2010) Validation of reference genes for quantitative real-time PCR during leaf and flower development in Petunia hybrida. BMC Plant Biol 10: 4. 20056000 10.1186/1471-2229-10-4 PMC2827423

[38] Podevin N, Krauss A, Henry I, Swennen R, Remy S (2012) Selection and validation of reference genes for quantitative RT-PCR expression studies of the non-model crop Musa. Mol Breed 30: 1237–1252. 23024595 10.1007/s11032-012-9711-1 PMC3460175

[39] Morgante CV, Guimaraes PM, Martins AC, Araujo AC, Leal-Bertioli SC, et al (2011) Reference genes for quantitative reverse transcription-polymerase chain reaction expression studies in wild and cultivated peanut. BMC Res Notes 4: 339. 21906295 10.1186/1756-0500-4-339 PMC3180468

[40] Marschner H (1995) Mineral nutrition of higher plants. London: Academic Press: 889.

[41] Epstein E, Bloom A (2005) Mineral nutrition of plants: principles and perspectives. 2nd edn Sunderland, MA: Sinauer Associates.

[42] Galloway JN, Cowling EB (2002) Reactive nitrogen and the world: 200 years of change. Ambio 31: 64–71. 12078011 10.1579/0044-7447-31.2.64

[43] Forde BG, Clarkson DT (1999) Nitrate and ammonium nutrition of plants: physiological and molecular perspectives. Advances in Botanical Research 30: 1–90.

[44] Maathuis F (2009) Physiological functions of mineral nutrients. Current Opinion in Plant Biology 12: 250–258. 19473870 10.1016/j.pbi.2009.04.003

[45] Crawford NM, Glass ADM (1998) Molecular and physiological aspects of nitrate uptake in plants. Trends in Plant Science 3: 389–395.

[46] Migocka M, Warzybok A, Kłobus G (2013) The genomic organization and transcriptional pattern of genes encoding nitrate transporters 1 (NRT1) in cucumber. Plant Soil 364: 245–260.

[47] Altschul SF, Madden TL, Schaffer AA, Zhang J, Zhang Z, et al (1997) Gapped BLAST and PSI-BLAST: a new generation of protein database search programs. Nucleic Acids Res 25: 3389–3402. 9254694 10.1093/nar/25.17.3389 PMC146917

[48] Salamov AA, Solovyev VV (2000) Ab initio gene finding in Drosophila genomic DNA. Genome Res 10: 516–522. 10779491 10.1101/gr.10.4.516 PMC310882

[49] Huang S, Li R, Zhang Z, Li L, Gu X, et al (2009) The genome of the cucumber, Cucumis sativus L. Nat Genet 41: 1275–1281. 19881527 10.1038/ng.475

[50] Woycicki R, Witkowicz J, Gawronski P, Dabrowska J, Lomsadze A, et al (2011) The genome sequence of the North-European cucumber (Cucumis sativus L.) unravels evolutionary adaptation mechanisms in plants. PLoS One 6: e22728. 21829493 10.1371/journal.pone.0022728 PMC3145757

[51] Matta BP, Bitner-Mathe BC, Alves-Ferreira M (2011) Getting real with real-time qPCR: a case study of reference gene selection for morphological variation in Drosophila melanogaster wings. Dev Genes Evol 221: 49–57. 21509536 10.1007/s00427-011-0356-6

[52] Hong SY, Seo PJ, Yang MS, Xiang F, Park CM (2008) Exploring valid reference genes for gene expression studies in Brachypodium distachyon by real-time PCR. BMC Plant Biol 8: 112. 18992143 10.1186/1471-2229-8-112 PMC2588586

[53] Huis R, Hawkins S, Neutelings G (2010) Selection of reference genes for quantitative gene expression normalization in flax (Linum usitatissimum L.). BMC Plant Biol 10: 71. 20403198 10.1186/1471-2229-10-71 PMC3095345

[54] Remans T, Smeets K, Opdenakker K, Mathijsen D, Vangronsveld J, et al (2008) Normalisation of real-time RT-PCR gene expression measurements in Arabidopsis thaliana exposed to increased metal concentrations. Planta 227: 1343–1349. 18273637 10.1007/s00425-008-0706-4

[55] Craig KL, Tyers M (1999) The F-box: a new motif for ubiquitin dependent proteolysis in cell cycle regulation and signal transduction. Prog Biophys Mol Biol 72: 299–328. 10581972 10.1016/s0079-6107(99)00010-3

[56] McMahon HT, Mills IG (2004) COP and clathrin-coated vesicle budding: different pathways, common approaches. Curr Opin Cell Biol 16: 379–391. 15261670 10.1016/j.ceb.2004.06.009

[57] Alberts B, Johnson A, Lewis J (2002) Transport into the Cell from the Plasma Membrane: Endocytosis. Molecular Biology of the Cell 4th edition New York: Garland Science.

[58] Ahn CS, Han JA, Lee HS, Lee S, Pai HS (2011) The PP2A regulatory subunit Tap46, a component of the TOR signaling pathway, modulates growth and metabolism in plants. Plant Cell 23: 185–209. 21216945 10.1105/tpc.110.074005 PMC3051261

[59] DeLong A (2006) Switching the flip: protein phosphatase roles in signaling pathways. Curr Opin Plant Biol 9: 470–477. 16890477 10.1016/j.pbi.2006.07.015

[60] Dheda K, Huggett JF, Bustin SA, Johnson MA, Rook G, et al (2004) Validation of housekeeping genes for normalizing RNA expression in real-time PCR. Biotechniques 37: 112–114, 116, 118–119. 15283208 10.2144/04371RR03

[61] Tenea GN, Peres Bota A, Cordeiro Raposo F, Maquet A (2011) Reference genes for gene expression studies in wheat flag leaves grown under different farming conditions. BMC Res Notes 4: 373. 21951810 10.1186/1756-0500-4-373 PMC3193821

[62] Hochstrasser M (1996) Protein degradation or regulation: Ub the judge. Cell 84: 813–815. 8601303 10.1016/s0092-8674(00)81058-2

