# Supplementary figures and images for: Reliable Reference Genes for Normalization of Gene Expression in Cucumber Grown under Different Nitrogen Nutrition
Source: PLoS One. 2013 Sep 13;8(9):e72887. doi: 10.1371/journal.pone.0072887 (PMC3772881; doi:10.1371/journal.pone.0072887)

SUPPORTING INFORMATION

Figure S1


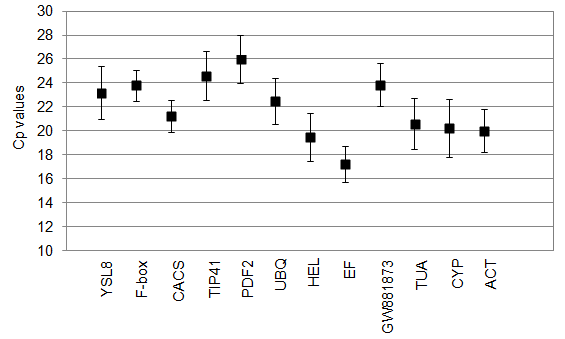

Supplement: Figure S1 — The average expression levels with SD of candidate reference genes in roots, stems and leaves of cucumber plants grown under different nitrogen nutrition. (DOC) [file pone.0072887.s001.doc]

SUPPORTING INFORMATION

Figure S2


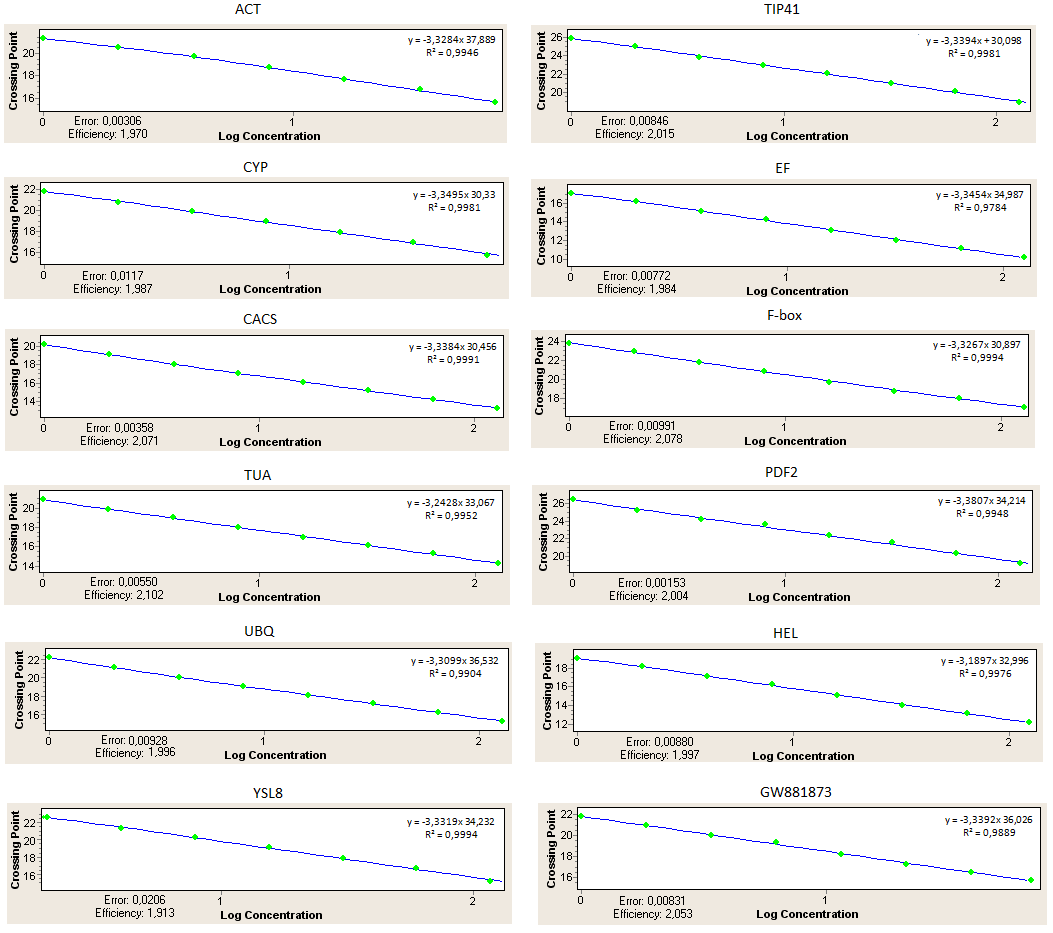

Supplement: Figure S2 — The parameters of real-time PCR amplification of candidate reference genes. (DOC) [file pone.0072887.s002.doc]

SUPPORTING INFORMATION

Figure S3


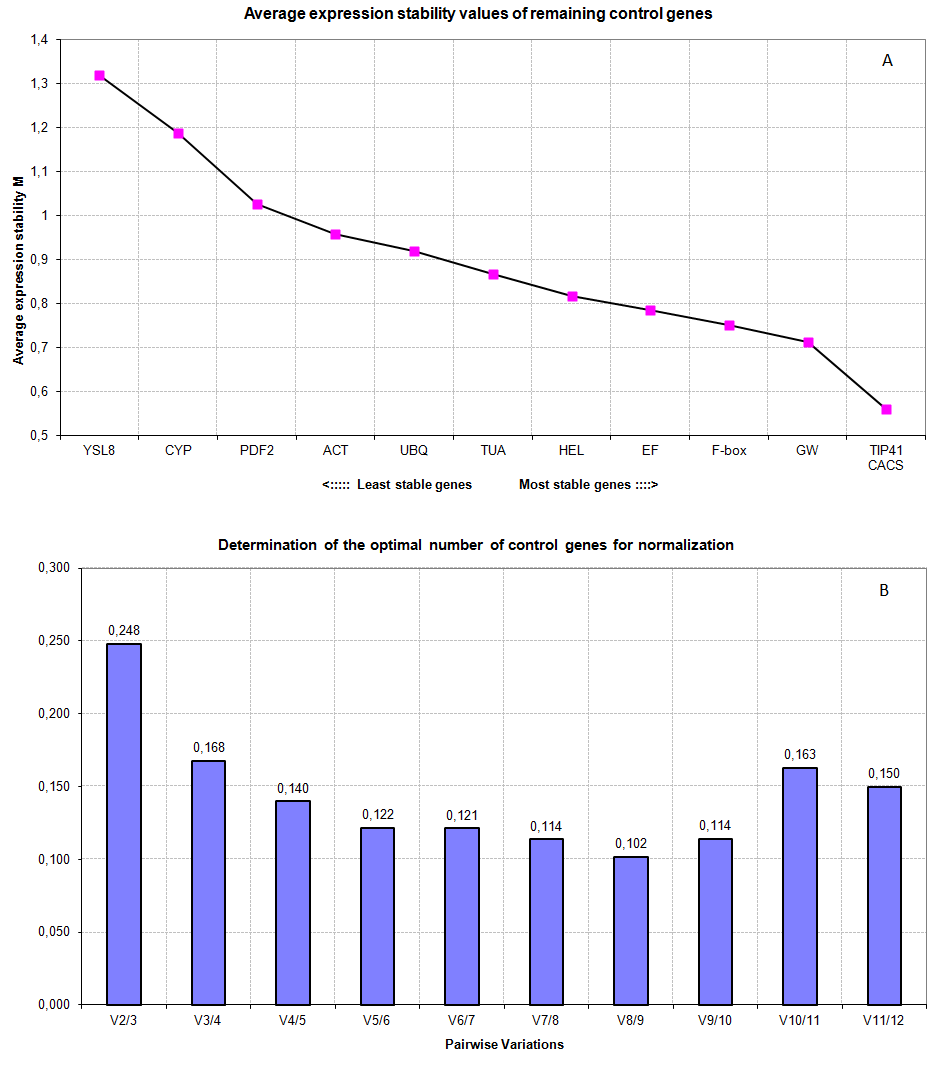

Supplement: Figure S3 — GeNorm based evaluation of candidate gene expression in samples from plants grown in different nitrogen compounds or under varying nitrate availability. A. Average expression stability values (M) of the remaining candidate cucumber reference genes during stepwise exclusion of the least stable reference gene all cucumbers organs. The lowest the M values indicate the most stable expression of candidate cucumber genes. B. Determination of optimal number of reference genes based on pairwise variation (V) analysis of normalization factors of the candidate reference genes all cucumber organs. The Vn/n+1 value was calculated for every comparison between two of the twelve consecutive candidate reference genes. According to [14], additional (n+1)th reference gene should be included into analysis whenever the Vn/n+1 value drops below the 0.15 threshold. (DOC) [file pone.0072887.s003.doc]
