# Supplementary material for: Reliable Reference Genes for Normalization of Gene Expression in Cucumber Grown under Different Nitrogen Nutrition
Source: PLoS One. 2013 Sep 13;8(9):e72887. doi: 10.1371/journal.pone.0072887 (PMC3772881; doi:10.1371/journal.pone.0072887)
Supplement: Table S1 — Primer sequences used to quantify the expression of the selected traditional and novel cucumber reference genes by real-time PCR. (DOC) [file pone.0072887.s004.doc]

SUPPORTING INFORMATION

Table S1

| Gene symbol | Forward primer sequence  (5'-3') | Reverse primer sequence  (5'-3') | Amplicon  length (bp) | Tm (°C) | PCR efficiency  (%) | Primer forward/reverse location |
| --- | --- | --- | --- | --- | --- | --- |
| *ACT* | CCGTTCTGTCCCTCTACGCTAGTG | GGAACTGCTCTTTGCAGTCTCGAG | 290 | 60 | 98.5 | exon2/exon3 |
| *TUA* | CATTCTCTCTTGGAACACACTGA | TCAAACTGGCAGTTAAAGATGAAA | 154 | 60 | 105.1 | exon4/exon5 |
| *EF α* | ACTTTATCAAGAACATGATTAC | TTCCTTCACAATTTCATCG | 230 | 60 | 99.2 | exon1/exon2 |
| *CYP* | GGAAATGGTACAGGAGGTG | CATACCCTCAACGACTTGAC | 201 | 60 | 99.35 | exon1/exon1 |
| *CACS* | GTGCTTTCTTTCTGGAATGC | TGAACCTCGTCAAATTTACACA | 158 | 60 | 103.55 | exon17/exon18 |
| *HEL* | TTCTCGAAGATTTAGTGATTCATGTG | CAATGGACGAATGCAAAGG | 168 | 60 | 99.85 | exon5/exon6 |
| *TIP41* | CAACAGGTGATATTGGATTATGATTATAC | GCCAGCTCATCCTCATATAAG | 221 | 60 | 100.75 | exon4/exon6 |
| *UBI-1* | CCTTATTGACCAACCAGTAGT | GGACAATGTTGATTTCCTCG | 164 | 60 | 99.8 | exon1/exon3 |
| *F-box* | GGTTCATCTGGTGGTCTT | CTTTAAACGAACGGTCAGTCC | 166 | 60 | 103.85 | exon1/exon1 |
| *YSL8* | CCTTGTGGATATCACAGAAGTT | CTTGTTTATCCTTGAGTGCC | 155 | 60 | 95.65 | exon2/exon2 |
| *GW881873* | GGGTCTAACTCATCATAAAGAAAGCG | CAGCATTCCATATGCTTATGTTCGT | 168 | 60 | 102.65 | exon 6/exon6 |
| *PDF2* | GTAGGACCTGAACCAACTA | CTTCACGCAGGGAAGA | 162 | 60 | 100.2 | exon4/exon5 |
| *CsNRT1.1* | GACAGGAACTATGCATTTGGGGAAT | GCGCAATGTGATGACGACTCTA | 240 | 60 | 100.5 | exon2/exon4 |
